# A Case Report of Recurrent Subacute Stent Thrombosis After Drug Eluting Stent Implantation: What Is the Real Reason?

**DOI:** 10.14740/cr448e

**Published:** 2015-12-16

**Authors:** Shi-Wei Yang, Yu-Jie Zhou

**Affiliations:** a Beijing Anzhen Hospital Affiliated to Capital Medical University; Beijing Institute of Heart, Lung and Blood Vessel Disease; The Key Laboratory of Remodeling-Related Cardiovascular Disease, Ministry of Education, Beijing, China

**Keywords:** Subacute stent thrombosis, Drug eluting stent, Stent edge dissection, Clopidogrel hyporesponse

## Abstract

A 63-year-old male was admitted with subacute anterior ST-elevation myocardial infarction. Cardiac catheterization revealed a subtotal occlusion in the proximal and middle part of left anterior descending coronary artery. Subacute stent thrombosis (SAT) occurred recurrently until the last stent deployment solved the problem of the uncovered artery and distal-stent edge dissection. The patient’s SYNTAX score was 19, and percutaneous coronary intervention (PCI) was performed. Unfortunately, SAT occurred recurrently after drug eluting stent implantation. What reason should be responsible for the recurrent SAT, clopidogrel hyporesponse or mechanical factors? All anti-platelet therapy has been tried, but adenosine diphosphate (ADP)-induced platelet aggregation remained hyporesponsive to clopidogrel. The patient has been symptom-free at follow-ups since the fourth PCI solved the problem of the uncovered artery and distal-stent edge dissection.

## Introduction

Stent thrombosis (ST) is a devastating complication of percutaneous coronary intervention (PCI) with significant morbidity and mortality. Despite a decreasing frequency of ST in the current era, these severe consequences have generated intense clinical and research interest in prevention and management. In the early period of bare metal stent (BMS), ST occurred in approximately 3-4% of patients despite aggressive anticoagulation regimens [1, 2]. Subsequent studies employing routine high-pressure dilation showed improved ST rates (< 1%) with dual anti-platelet therapy (DAPT) compared to systemic anticoagulation [3]. Though the focus has shifted to late ST since the BASKET-LATE trial being reported, most series reporting drug eluting stent (DES) thrombosis have shown that acute or subacute stent thrombosis (SAT) still outnumbers late events and hence remains an important entity [4].

## Case Report

**Figure 1:**
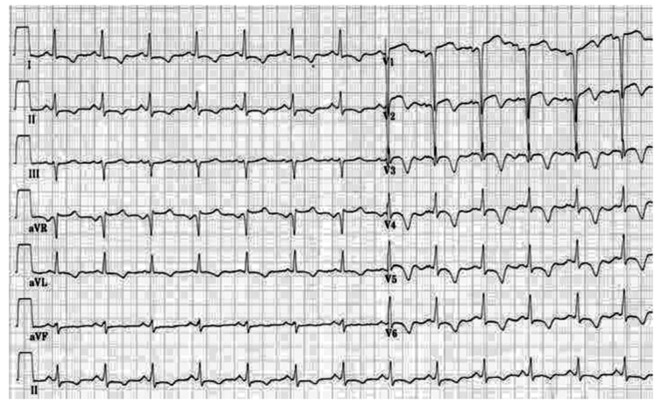
Electrocardiogram (ECG) on admission demonstrated normal sinus rhythm and anterior precordial leads ST-segment elevation and T waves inversion.

**Figure 2:**
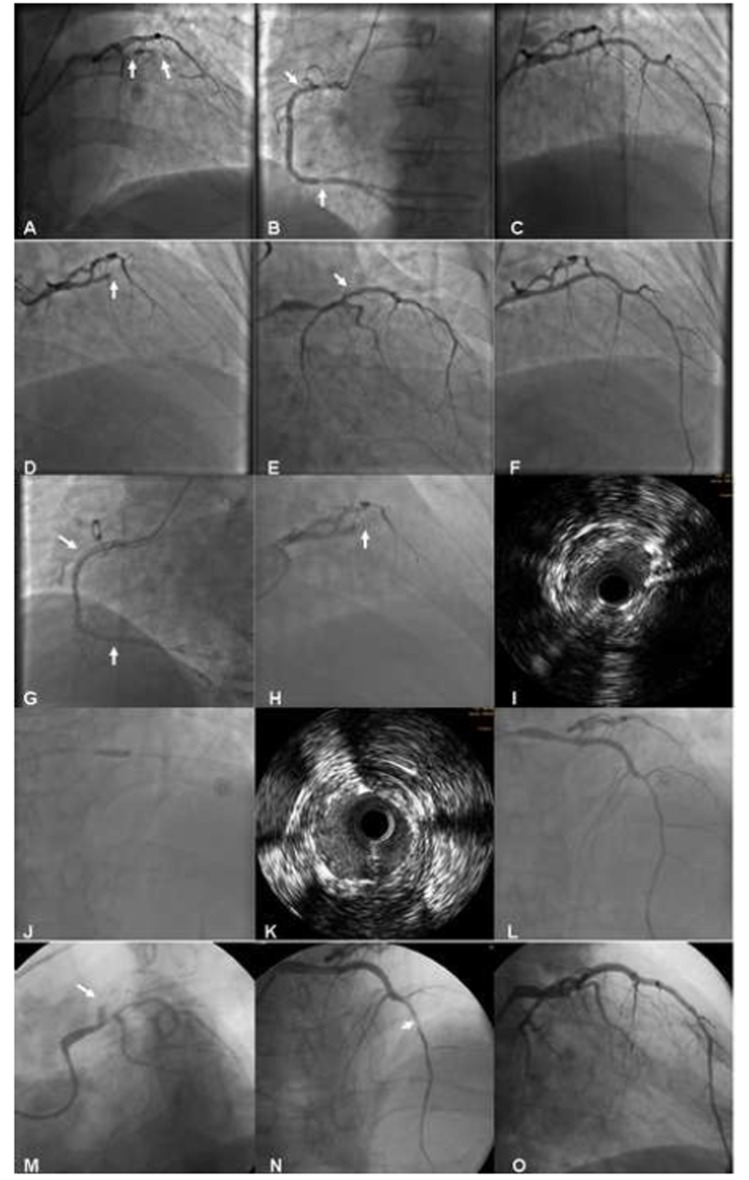
Images in the course of all procedures.

A 63-year-old male was admitted after sustaining an ST-elevation myocardial infarction (STEMI) just 10 days ago. Cardiovascular risk factors included diabetes mellitus type 2, hypertension, hyperlipidemia and smoking. Despite aggressive medical therapy, frequent rest angina and short of breath recurred at the time of admission to our hospital. Physical examination revealed fine wet rales in the bottom part of the lung. Laboratory values showed troponin I (TnI) of 1.17 ng/mL (normal range 0 - 0.05 ng/mL), low-density lipoprotein cholesterol of 4.6 mmol/L, creatinine of 93 μmol/L, fasting plasma glucose of 9.64 mmol/L, hemoglobin A1c (HbA1c) of 6.7%, and brain natriuretic peptide (BNP) of 1,958.5 pg/mL (normal range 0 - 125 pg/mL). Electrocardiogram (ECG) demonstrated normal sinus rhythm and anterior precordial leads ST-segment elevation and T waves inversion (Fig. 1). An echocardiogram revealed an enlarged left ventricular cavity (left ventricular end-diastolic dimension 61 mm, left ventricular end-systolic dimension 42 mm) and a nearly normal left ventricular ejection fraction (LVEF) of 53% with segmental wall-motion abnormalities: hypokinesis in anterior wall and the septum. The diagnosis of subacute anterior STEMI was made based on the history and clinical examination. Cardiac catheterization revealed a subtotal occlusion in the proximal and middle part of left anterior descending coronary artery (LAD), mild stenosis in the left main coronary artery (LM) (Fig. 2A) and diffuse lesion in the proximal and 85% stenosis in the distal part of right coronary artery (RCA) (Fig. 2B). According to the patient’s SYNTAX score of 22, we decided to perform PCI for these lesions.

After predilation with 1.5 × 15 mm and 2.0 × 20 mm Ryujin balloons (Terumo, Tokyo, Japan), two sirolimus-eluting stents (SES) were implanted successfully: Firebird (MicroPort Medical, Shanghai, China) 2.75 × 23 mm in middle LAD and Excel (JW Medical, Shandong, China) 3.0 × 24 mm in the proximal LAD (2 to 3-mm overlap at the ends). Post-dilation was performed with a non-compliance 3.0 × 24 mm Maverick Quantum balloon (Boston Scientific, Boston, MA). TIMI-3 flow was noted and no significant residual stenosis remained (Fig. 2C). After the procedure, daily oral DAPT of clopidogrel 75 mg and aspirin (ASA) 300 mg were prescribed. Glycoprotein IIb/IIIa receptor antagonist tirofiban was initially given as rapid intravenous infusion at a rate of 0.4 μg/kg/min for 30 min. Upon completion of the initial infusion, the rate is decreased to 0.15 μg/kg/min delivered as continuous infusion for about 36 h.

Approximately 2 h after the end of tirofiban administration, the patient developed severe chest pain and T-wave pseudonormalization in the anterior leads on ECG. He was immediately moved to the catheterization lab for repeat coronary angiography (CAG) and ST was found in LAD (Fig. 2D). In consideration of the possibility of a dissection at the proximal stent edge (Fig. 2E), an additional SES of Partner 3.0 × 21 mm (Lepu Medical, Beijing, China) was deployed, overlapping the previous proximal stent edge, with an excellent final result (Fig. 2F). Then, Partner 4.0 × 15 mm (Lepu Medical, Beijing, China) and Taxus 4.0 × 38 mm (Boston Scientific, Natick, MA) were implanted in the distal and proximal segment of RCA, respectively (Fig. 2G). The patient’s clopidogrel dose was increased to 75 mg twice a day because he was found to be hyporesponsive to clopidogrel when tested for adenosine diphosphate (ADP)-induced platelet aggregation utilizing with light transmission aggregometry (LTA). He did not taking any drugs that could deteriorate the platelet function, including proton pump inhibitor. Tirofiban was given as continuous infusion for about 72 h. The patient had an uneventful recovery in the following 5 days.

**Figure 3:**
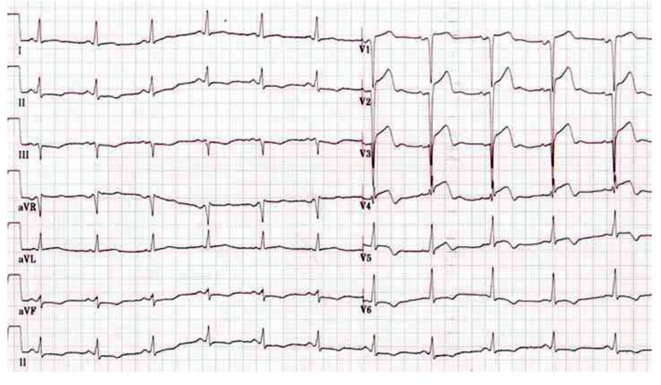
ECG showed ST-segment elevation in V1-V5 leads when the patient again underwent severe chest pain in the sixth day after the second PCI.

Unfortunately, the patient again underwent severe chest pain in the sixth day after the second PCI, and the ECG showed ST-segment elevation in V1-V5 leads (Fig. 3). Misgiving about the uncertainty of the reason for recurrent ST, surgeons refused to perform coronary artery bypass grafting (CABG). After failure of initial thrombolytic therapy, he underwent emergent salvage angiography by a different interventionalist. Emergency CAG showed total occlusion of the mid-LAD due to ST (Fig. 2H). Thrombi were aspirated with the Diver CE aspiration catheter (Invatec, Brescia, Italy). Intravascular ultrasound (IVUS) revealed inadequate stent expansion due to elastic recoil (Fig. 2I). This was repaired by repeated high-pressure post-dilation with 3.0 × 12 mm and 3.5 × 15 mm NC Sprinter balloons (Medtronic, Minneapolis, MN) (Fig. 2J, K, L). Despite the double dose of clopidogrel therapy, ADP-induced platelet aggregation showed that she was still hyporesponsive to clopidogrel. The patient had an uncomplicated recovery and was discharged 1 week later with triple anti-platelet therapy (aspirin 300 mg qd, clopidogrel 75 mg bid, and cilostazol 100 mg bid).

The patient’s chest pain recurred again 6 days thereafter. Repeat emergent CAG revealed patent RCA stents and total occlusion of the mid-LAD due to ST (Fig. 2M). Surgeons still refused to perform CABG for this patient. Diver CE aspiration catheter (Invatec, Brescia, Italy) was used again. Given the possibility of a dissection at the distal stent edge (Fig. 2N), we deployed another SES of Firebird (MicroPort Medical, Shanghai, China) 2.5 × 29 mm in distal-LAD, overlapping the previous distal stent edge. The final result revealed no residual stenosis or dissection and excellent TIMI-3 flow (Fig. 2O). Upon completion of continuous infusion of tirofiban for about 7 days, the patient was discharged. He was hemodynamically stable and symptom-free at the time of discharge. Echocardiogram revealed a decreased LVEF of 48%. Laboratory retest showed low-density lipoprotein cholesterol of 1.6 mmol/L, creatinine of 96 μmol/L, and fasting plasma glucose of 6.02 mmol/L. Although the patient has complied strictly with the triple anti-platelet therapy (aspirin 300 mg qd, clopidogrel 75 mg bid, and cilostazol 100 mg bid), ADP-induced platelet aggregation showed that she was still hyporesponsive to clopidogrel. Given the very complicated recent course, no further interventions were undertaken. Fortunately, the patient has been symptom-free at follow-ups.

## Discussion

Though the focus has shifted to late ST since the BASKET-LATE trial being reported, most series reporting DES thrombosis have shown that acute or SAT still outnumbers late events and hence remains an important entity [4]. We report this case just to draw more attention to the clinical features, mechanisms and management of SAT in the DES era. ST was classified by the Academic Research Consortium (ARC) definition as definite, probable, or possible and as acute (0 to 24 h), subacute (1 to 30 days), late (31 to 360 days), and very late (> 360 days) [5]. SAT was an initial major limitation of BMS implantation [6]. SAT was reported to have an incidence of 0.9% in the modern BMS era [7]. Features on IVUS found to be associated with SAT included stent under-expansion, malapposition, inflow/outflow disease, dissection, thrombus, and tissue prolapse [6, 8, 9]. These findings confirmed that the mechanisms underlying SAT were mechanical and potentially treatable when identified. It is reasonable to assume that the underlying mechanisms of SAT after both implantation of BMS and DES remain the same, predominantly mechanical.

High-pressure stent deployment has become standard practice to produce the greatest post-procedure minimal luminal diameter in the target vessel. Maximizing the acute gain in lumen size achieved by stent deployment could significantly offset some of the late loss produced by intimal proliferation and reduce the risk of SAT [10]. High-pressure balloon inflation within the stent reliably opposes the device struts to the vessel wall without increasing the risk of major disruptions of vessel architecture (hematomas, dissections, and rupture) within the stented segments [11]. However, more aggressive deployment of stents has resulted in dissections at the transition between the stented segment and the uncovered artery, termed edge dissections, marginal dissections, or pocket tears, in 5-23% of cases when examined with IVUS [12]. Before the use of IVUS, many of these edge dissections went undetected angiographically. However, a negative IVUS result could not absolutely eliminate the possibility of mild edge dissection, just as this case told us. DAPT with aspirin and a thienopyridine, most commonly clopidogrel, is generally recommended after stent implantation to mitigate the risk of ST. Recent studies have shown that adequate anti-platelet effects are not achieved in 5-45% of the patients taking aspirin and in 4-30% of patients taking clopidogrel and therefore suggest that many patients are hyporesponsive to the anti-platelet agents [13].

Currently, however, routine screening for anti-platelet hyporesponse remains a persistent, unresolved issue and further evidence is necessary before it will be possible to recommend this testing as part of standard assessment of PCI candidates. The treatment of anti-platelet hyporesponse is as yet undefined. Several therapeutic approaches (increased dosage of clopidogrel and aspirin, the addition of cilostazol or a glycoprotein IIb/IIIa inhibitor, etc.) might be prescribed [14]. In our patient, all above have been tried, but ADP-induced platelet aggregation utilizing with LTA was still hyporesponsive to clopidogrel. And SAT occurred recurrently until the last stent deployment solved the problem of the uncovered artery and distal-stent edge dissection. Accordingly, clopidogrel hyporesponse may be a comorbidity, but not the main reason for recurrent SAT.

### Conclusions

SAT remains an important catastrophic complication in the DES era. The mechanisms underlying SAT were mainly mechanical and potentially treatable when identified in the early stage. One should be cautious to draw a conclusion that anti-platelet hyporesponse is responsible for SAT. Currently, diagnosis, prevention and treatment of anti-platelet hyporesponse are all unresolved.
